# The genome sequence of the hazel dormouse, *Muscardinus avellanarius *(Linnaeus, 1758)

**DOI:** 10.12688/wellcomeopenres.20360.1

**Published:** 2023-11-10

**Authors:** Astrid Böhne, Christine Thiel-Bender, Sandra Kukowka, Darwin Tree of Life Barcoding collective, Wellcome Sanger Institute Tree of Life programme, Wellcome Sanger Institute Scientific Operations: DNA Pipelines collective, Tree of Life Core Informatics collective, Darwin Tree of Life Consortium

**Affiliations:** 1 Centre for Molecular Biodiversity Research, Leibniz Institute for the Analysis of Biodiversity Change,, Museum Koenig Bonn, Bonn, Germany; 2 Bund für Umwelt und Naturschutz Deutschland (BUND) Landesverband NRW e.V.,, Friends of the Earth Germany, Düsseldorf, Germany

**Keywords:** Muscardinus avellanarius, hazel dormouse, genome sequence, chromosomal, Rodentia

## Abstract

We present a genome assembly from an individual male *Muscardinus avellanarius* (the hazel dormouse; Chordata; Mammalia; Rodentia; Gliridae). The genome sequence is 2,497.5 megabases in span. Most of the assembly is scaffolded into 24 chromosomal pseudomolecules, including the X and Y sex chromosomes. The mitochondrial genome has also been assembled and is 16.73 kilobases in length.

## Species taxonomy

Eukaryota; Metazoa; Eumetazoa; Bilateria; Deuterostomia; Chordata; Craniata; Vertebrata; Gnathostomata; Teleostomi; Euteleostomi; Sarcopterygii; Dipnotetrapodomorpha; Tetrapoda; Amniota; Mammalia; Theria; Eutheria; Boreoeutheria; Euarchontoglires; Glires; Rodentia; Sciuromorpha; Gliridae; *Muscardinus, Muscardinus avellanarius* (Linnaeus, 1758) (NCBI:txid39082).

## Background

The hazel or common dormouse, *Muscardinus avellanarius* Linnaeus 1758, is brown to ochre-coloured and has a furred tail. It can be as small as 7 cm, and lives arboreally in forests of all types and age classes, including pure spruce forests, park landscapes and riverine forests as well as hedges and shrubberies. The animals hibernate in self-constructed nests on the ground or between rootstocks, and sometimes in nestboxes. The hazel dormouse feeds on seeds, flower buds, berries and other fruits, and nuts, especially hazelnuts. Hazel dormice have a mostly vegetarian diet, but in early summer up to 50 % of the food can consist of insects. It is the only extant species of the genus *Muscardinu*s.

The hazel dormouse is found in northern Europe and Asia Minor and is strictly protected in Europe under the Habitat Directive (annex IV) and Bern Convention (annex III). It is the only dormouse native to Britain, where it is protected under the Wildlife and Countryside Act since its reintroduction in 1993 as part of the English Nature Species Recovery Programme. It is an introduced species to Ireland. In the 2006 European report ( Kryštufek *et al.*, 2007) as well as the 2016 global assessment ( Hutterer *et al.*, 2021) of the IUCN Red List of Threatened Species, the hazel dormouse is listed as ‘least concern’. The German Red List has the hazel dormouse as ‘near threatened’, with current observations being rare and a moderate declining trend in the long-term in the population ( Meinig *et al.*, 2020). The hazel dormouse is threatened by woodland habitat loss and the resulting fragmentation as well as changes in woodland management practices since it depends on forests in combination with hedges and bushes, as dormice do not cross large, open spaces.

Based on mitochondrial markers, Mouton *et al*. (2012) identified two highly divergent *M. avellanarius* lineages (lineage 1 in France, Belgium, Switzerland and Italy, lineage 2 in Poland, Germany, Latvia, Lithuania, the Balkan Peninsula and Turkey) and low genetic diversity within the lineages. This underlines the importance of further genomic studies to define conservation units for this species.

Here we present a chromosomally complete genome sequence for *Muscardinus avellanarius* based on one male specimen from Friesheimer Busch, Germany.

## Genome sequence report

**Figure 1.**
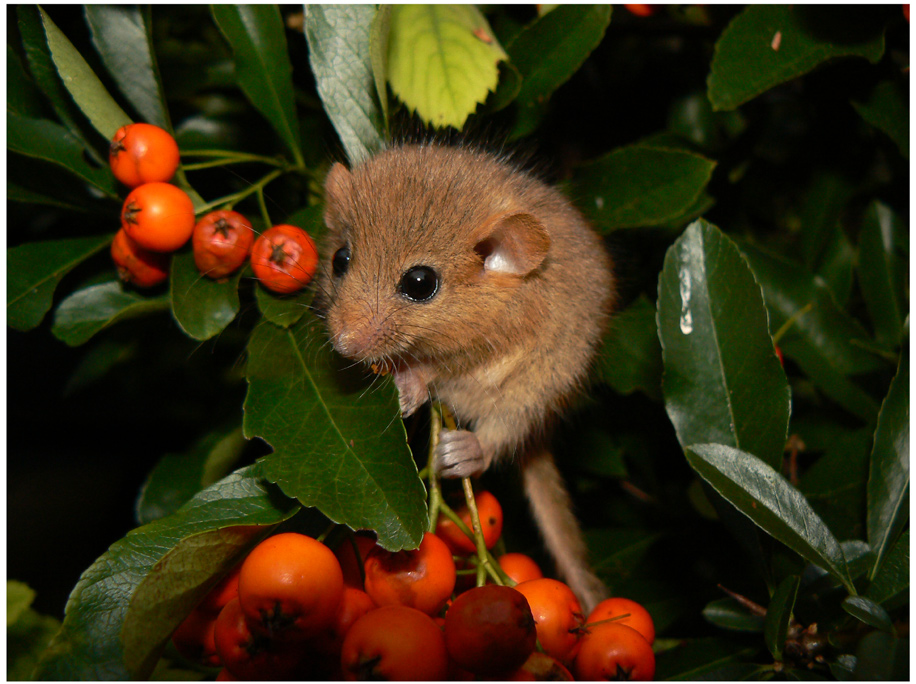
Hazel dormouse ( *Muscardinus avellanarius*) on a Firethorn (Pyracantha coccinea) by Danielle Schwarz (CC-BY).

The genome was sequenced from one male *Muscardinus avellanarius* ( Figure 1) collected from Friesheimer Busch, Germany. A total of 58-fold coverage in Pacific Biosciences single-molecule HiFi long reads was generated. Primary assembly contigs were scaffolded with chromosome conformation Hi-C data. Manual assembly curation corrected 34 missing joins or mis-joins, reducing the scaffold number by 6.58%.

**Figure 2.**
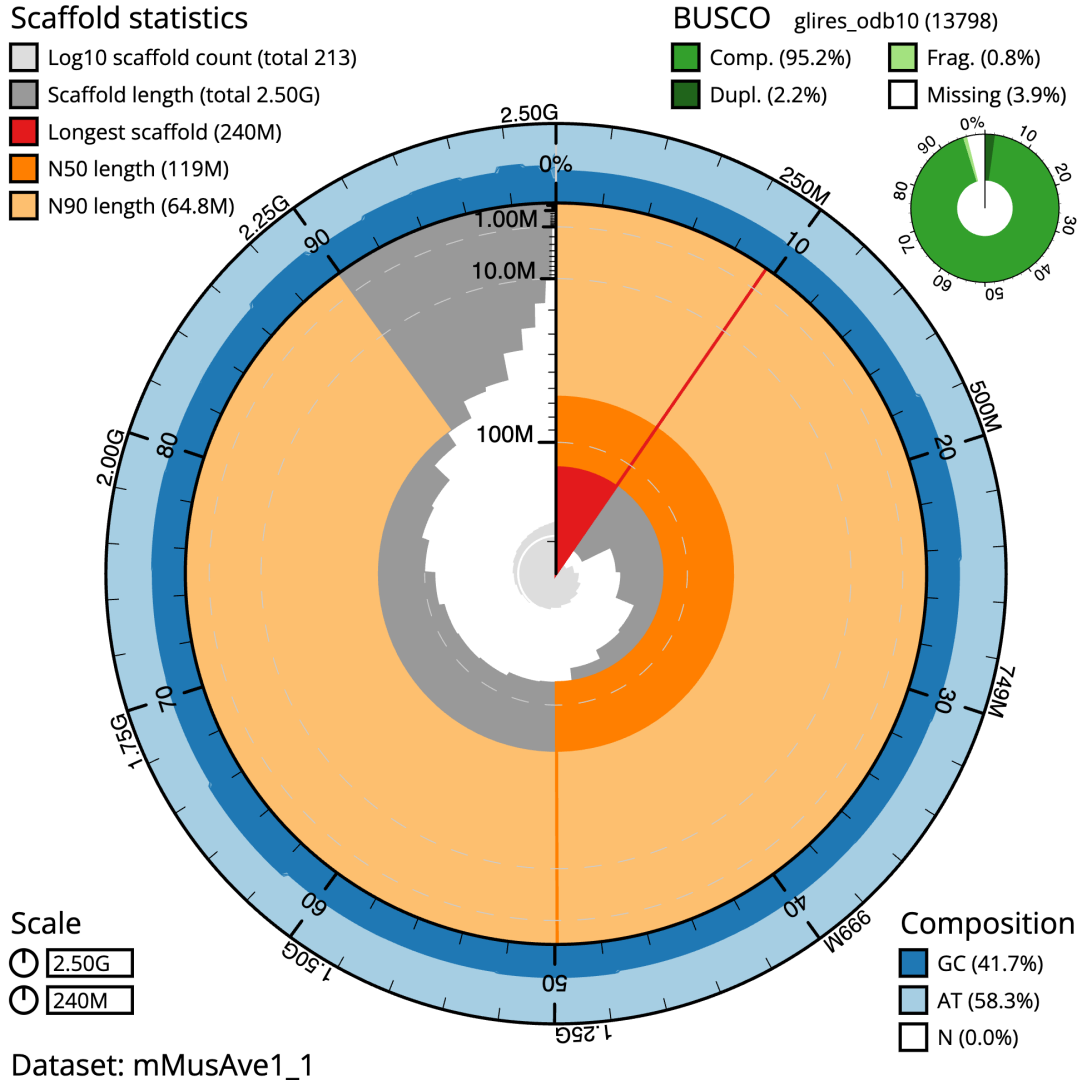
Genome assembly of *Muscardinus avellanarius*, mMusAve1.1: metrics. The BlobToolKit Snailplot shows N50 metrics and BUSCO gene completeness. The main plot is divided into 1,000 size-ordered bins around the circumference with each bin representing 0.1% of the 2,497,520,702 bp assembly. The distribution of scaffold lengths is shown in dark grey with the plot radius scaled to the longest scaffold present in the assembly (239,886,027 bp, shown in red). Orange and pale-orange arcs show the N50 and N90 scaffold lengths (119,481,734 and 64,769,431 bp), respectively. The pale grey spiral shows the cumulative scaffold count on a log scale with white scale lines showing successive orders of magnitude. The blue and pale-blue area around the outside of the plot shows the distribution of GC, AT and N percentages in the same bins as the inner plot. A summary of complete, fragmented, duplicated and missing BUSCO genes in the glires_odb10 set is shown in the top right. An interactive version of this figure is available at https://blobtoolkit.genomehubs.org/view/Muscardinus%20avellanarius/dataset/mMusAve1_1/snail.

**Figure 3.**
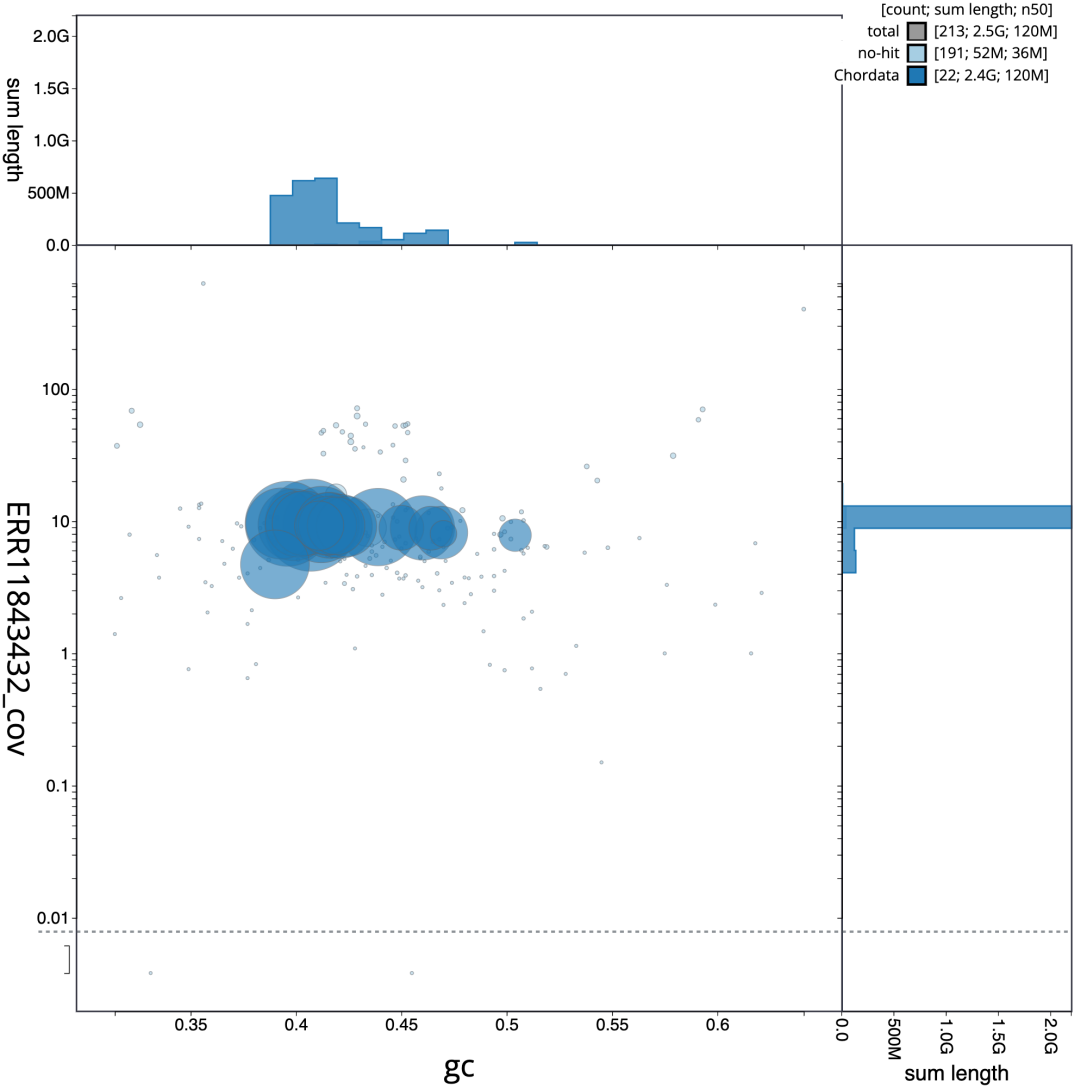
Genome assembly of *Muscardinus avellanarius*, mMusAve1.1: BlobToolKit GC-coverage plot. Scaffolds are coloured by phylum. Circles are sized in proportion to scaffold length. Histograms show the distribution of scaffold length sum along each axis. An interactive version of this figure is available at https://blobtoolkit.genomehubs.org/view/Muscardinus%20avellanarius/dataset/mMusAve1_1/blob.

**Figure 4.**
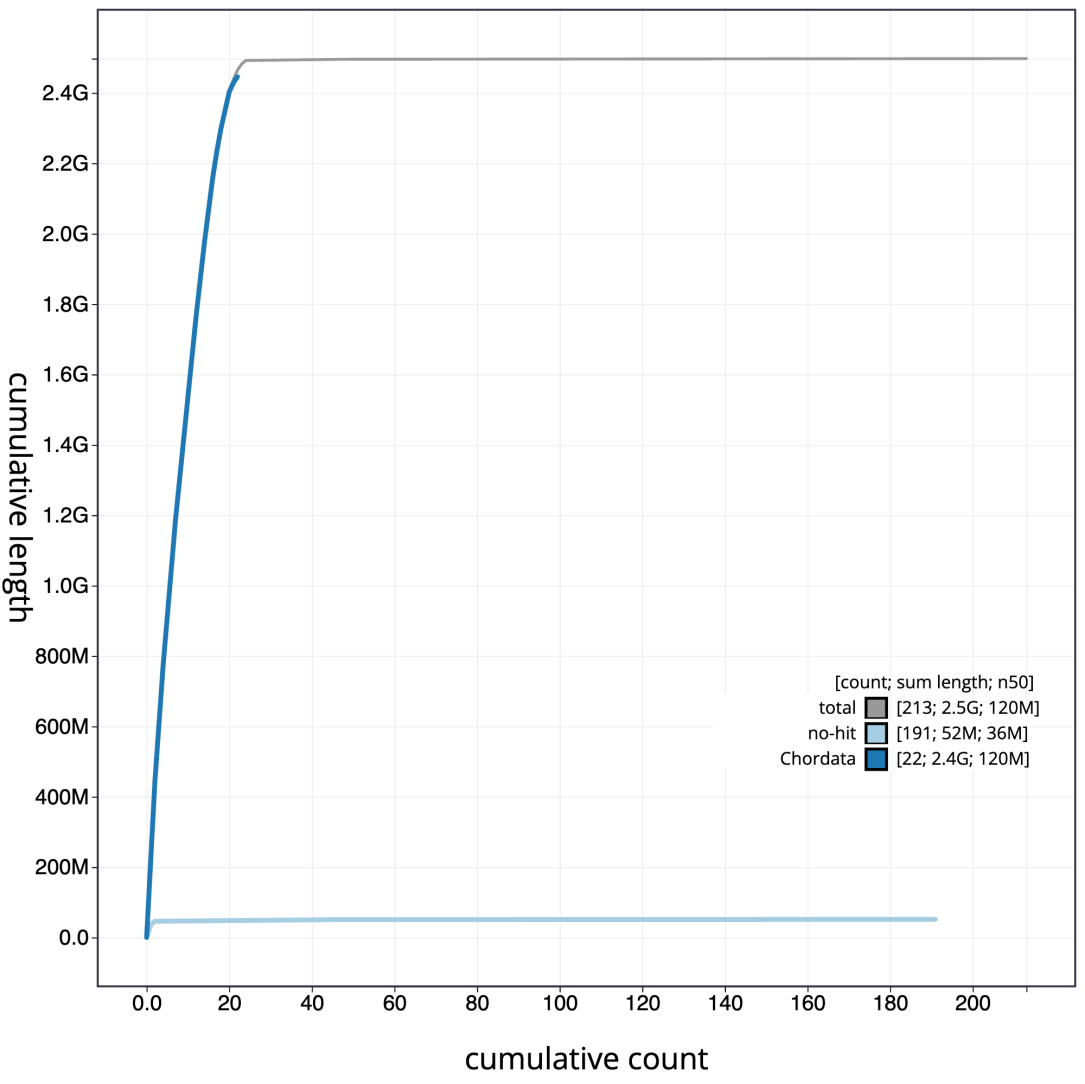
Genome assembly of *Muscardinus avellanarius*, mMusAve1.1: BlobToolKit cumulative sequence plot. The grey line shows cumulative length for all scaffolds. Coloured lines show cumulative lengths of scaffolds assigned to each phylum using the buscogenes taxrule. An interactive version of this figure is available at https://blobtoolkit.genomehubs.org/view/Muscardinus%20avellanarius/dataset/mMusAve1_1/cumulative.

**Figure 5.**
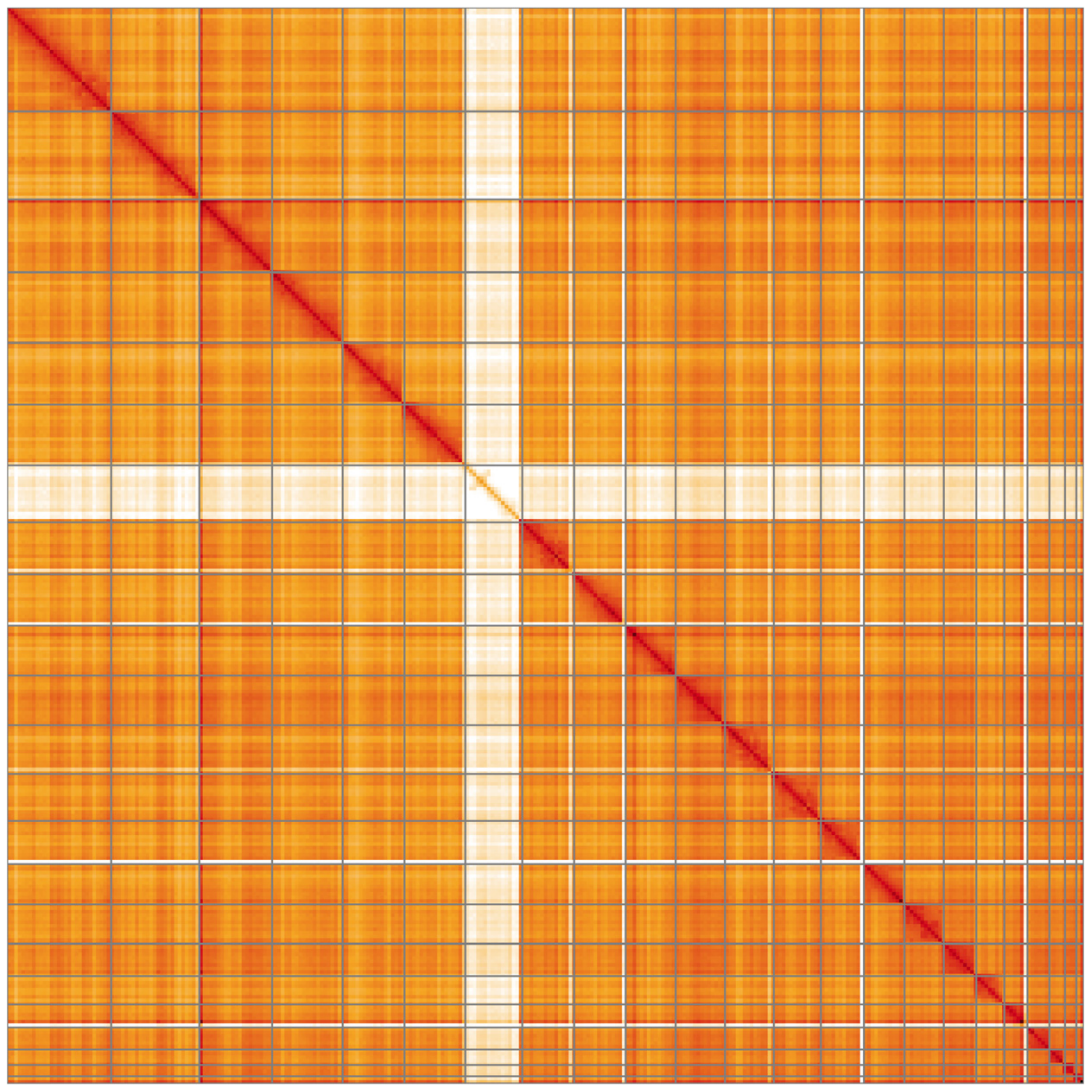
Genome assembly of *Muscardinus avellanarius*, mMusAve1.1: Hi-C contact map of the mMusAve1.1 assembly, visualised using HiGlass. Chromosomes are shown in order of size from left to right and top to bottom. An interactive version of this figure may be viewed at https://genome-note-higlass.tol.sanger.ac.uk/l/?d=U7gYOKXVRseTzqyRCHHU5g.

The final assembly has a total length of 2497.5 Mb in 212 sequence scaffolds with a scaffold N50 of 119.5 Mb ( Table 1). The snailplot in Figure 2 provides a summary of the assembly statistics, while the distribution of assembly scaffolds on GC proportion and coverage is shown in Figure 3. The cumulative assembly plot in Figure 4 shows curves for subsets of scaffolds assigned to different phyla. Most (99.78%) of the assembly sequence was assigned to 24 chromosomal-level scaffolds, representing 22 autosomes and the X and Y sex chromosomes. Chromosome-scale scaffolds confirmed by the Hi-C data are named in order of size ( Figure 5; Table 2). While not fully phased, the assembly deposited is of one haplotype. Contigs corresponding to the second haplotype have also been deposited. The mitochondrial genome was also assembled and can be found as a contig within the multifasta file of the genome submission.

**Table 1.** Genome data for *Muscardinus avellanarius*, mMusAve1.1.

Project accession data		
Assembly identifier	mMusAve1.1	
Assembly release date	2023-08-26	
Species	*Muscardinus avellanarius*	
Specimen	mMusAve1	
NCBI taxonomy ID	39082	
BioProject	PRJEB64959	
BioSample ID	SAMEA110180654	
Isolate information	mMusAve1, male (DNA sequencing and Hi-C data)	
Assembly metrics *		*Benchmark*
Consensus quality (QV)	53.7	*≥ 50*
*k*-mer completeness	99.98	*≥ 95%*
BUSCO **	C:96.2%[S:93.4%,D:2.9%], F:0.6%,M:3.1%,n:13,798	*C ≥ 95%*
Percentage of assembly mapped to chromosomes	99.78%	*≥ 95%*
Sex chromosomes	X and Y chromosomes	*localised homologous pairs*
Organelles	Mitochondrial genome assembled	*complete single alleles*
Raw data accessions		
PacificBiosciences SEQUEL II	ERR11843432	
Hi-C Illumina	ERR11837521	
Genome assembly		
Assembly accession	GCA_963383645.1	
*Accession of alternate haplotype*	GCA_963383665.1	
Span (Mb)	2497.5	
Number of contigs	2018	
Contig N50 length (Mb)	2.4	
Number of scaffolds	212	
Scaffold N50 length (Mb)	119.5	
Longest scaffold (Mb)	239.9	

* Assembly metric benchmarks are adapted from column VGP-2020 of “Table 1: Proposed standards and metrics for defining genome assembly quality” from ( Rhie *et al.*, 2021). ** BUSCO scores based on the glires_odb10 BUSCO set using v5.3.2. C = complete [S = single copy, D = duplicated], F = fragmented, M = missing, n = number of orthologues in comparison. A full set of BUSCO scores is available at https://blobtoolkit.genomehubs.org/view/Muscardinus%20avellanarius/dataset/mMusAve1_1/busco.

**Table 2.** Chromosomal pseudomolecules in the genome assembly of *Muscardinus avellanarius*, mMusAve1.

INSDC accession	Chromosome	Length (Mb)	GC%
OY726557.1	1	239.89	40.5
OY726558.1	2	203.09	39.5
OY726559.1	3	167.7	44.0
OY726560.1	4	162.78	41.0
OY726561.1	5	142.52	40.0
OY726562.1	6	140.28	39.5
OY726564.1	7	119.48	41.5
OY726565.1	8	118.69	40.0
OY726566.1	9	115.13	40.5
OY726567.1	10	113.83	46.0
OY726568.1	11	112.81	42.0
OY726569.1	12	108.21	41.5
OY726570.1	13	99.45	42.5
OY726571.1	14	93.12	41.5
OY726572.1	15	90.84	42.0
OY726573.1	16	74.91	47.0
OY726574.1	17	64.77	41.0
OY726575.1	18	53.12	45.0
OY726576.1	19	50.66	46.5
OY726577.1	20	36.0	43.5
OY726578.1	21	26.22	50.5
OY726579.1	22	17.06	47.0
OY726563.1	X	131.3	39.0
OY726580.1	Y	10.19	42.0
OY726581.1	MT	0.02	35.5

The estimated Quality Value (QV) of the final assembly is 53.7 with *k*-mer completeness of 99.98%, and the assembly has a BUSCO v5.3.2 completeness of 96.2% (single = 93.4%, duplicated = 2.9%), using the glires_odb10 reference set ( *n* = 13,798).

Metadata for specimens, barcode results, spectra estimates, sequencing runs, contaminants and pre-curation assembly statistics are given at https://links.tol.sanger.ac.uk/species/39082.

## Methods

### Sample acquisition and nucleic acid extraction

The sequenced individual *Muscardinus avellanarius* (specimen ID SAN00002415, ToLID mMusAve1) was found dead in Friesheimer Busch, Germany on 2021-07-21 and then frozen in a regular –20 °C freezer. Astrid Böhne, Christine Thiel-Bender and Sandra Kukowka collected and identified the species. Subsamples for genome sequencing were taken from the frozen individual with minimal thawing and transferred to dry ice and subsequent storage at –80 °C until further processing. The appropriation of a dead found hazel dormouse and subsequent usage for research was granted by the responsible authority Untere Naturschutzbehörde Rhein-Erft Kreis as an exemption to §45 ABS4 Bundesnaturschutzgesetz Germany.

The workflow for high molecular weight (HMW) DNA extraction at the Wellcome Sanger Institute (WSI) includes a sequence of core procedures: sample preparation; sample homogenisation; DNA extraction; HMW DNA fragmentation; and fragmented DNA clean-up. The mMusAve1 sample was weighed and dissected on dry ice with tissue set aside for Hi-C sequencing (as per the protocol at https://dx.doi.org/10.17504/protocols.io.x54v9prmqg3e/v1). For sample homogenisation, thorax tissue was cryogenically disrupted using the Sample Homogenisation: Covaris cryoPREP® Automated Dry Pulverizer protocol ( https://dx.doi.org/10.17504/protocols.io.eq2lyjp5qlx9/v1). HMW DNA was extracted by means of the Automated MagAttract protocol ( https://dx.doi.org/10.17504/protocols.io.kxygx3y4dg8j/v1). HMW DNA was sheared into an average fragment size of 12–20 kb in a Megaruptor 3 system with speed setting 30, following the HMW DNA Fragmentation: Diagenode Megaruptor®3 for PacBio HiFi protocol ( https://dx.doi.org/10.17504/protocols.io.8epv5x2zjg1b/v1). Sheared DNA was purified following either the Manual solid-phase reversible immobilisation (SPRI) protocol ( https://dx.doi.org/10.17504/protocols.io.kxygx3y1dg8j/v1), or the Automated SPRI protocol ( https://dx.doi.org/10.17504/protocols.io.q26g7p1wkgwz/v1) for higher throughput. In brief, the method employs a 1.8X ratio of AMPure PB beads to sample to eliminate shorter fragments and concentrate the DNA. The concentration of the sheared and purified DNA was assessed using a Nanodrop spectrophotometer and Qubit Fluorometer and Qubit dsDNA High Sensitivity Assay kit. Fragment size distribution was evaluated by running the sample on the FemtoPulse system.

Protocols employed by the Tree of Life laboratory are publicly available on protocols.io: https://dx.doi.org/10.17504/protocols.io.8epv5xxy6g1b/v1.

### Sequencing

Pacific Biosciences HiFi circular consensus DNA sequencing libraries were constructed according to the manufacturers’ instructions. DNA sequencing was performed by the Scientific Operations core at the WSI on a Pacific Biosciences SEQUEL II (HiFi) instrument. Hi-C data were also generated from tissue of mMusAve1 using the Arima2 kit and sequenced on the Illumina NovaSeq 6000 instrument.

### Genome assembly, curation and evaluation

Assembly was carried out with Hifiasm ( Cheng *et al.*, 2021) and haplotypic duplication was identified and removed with purge_dups ( Guan *et al.*, 2020). The assembly was then scaffolded with Hi-C data ( Rao *et al.*, 2014) using YaHS ( Zhou *et al.*, 2023). The assembly was checked for contamination and corrected using the using the TreeVal pipeline ( Pointon *et al.*, 2022). Manual curation was performed using JBrowse2 ( Diesh *et al.*, 2023), HiGlass ( Kerpedjiev *et al.*, 2018) and Pretext ( Harry, 2022). The mitochondrial genome was assembled using MitoHiFi ( Uliano-Silva *et al.*, 2023), which runs MitoFinder ( Allio *et al.*, 2020) or MITOS ( Bernt *et al.*, 2013) and uses these annotations to select the final mitochondrial contig and to ensure the general quality of the sequence.

A Hi-C map for the final assembly was produced using bwa-mem2 ( Vasimuddin *et al.*, 2019) in the Cooler file format ( Abdennur & Mirny, 2020). To assess the assembly metrics, the *k*-mer completeness and QV consensus quality values were calculated in Merqury ( Rhie *et al.*, 2020). This work was done using Nextflow ( Di Tommaso *et al.*, 2017) DSL2 pipelines “sanger-tol/readmapping” ( Surana *et al.*, 2023a) and “sanger-tol/genomenote” ( Surana *et al.*, 2023b). The genome was analysed within the BlobToolKit environment ( Challis *et al.*, 2020) and BUSCO scores ( Manni *et al.*, 2021; Simão *et al.*, 2015) were calculated.

Table 3 contains a list of relevant software tool versions and sources.

**Table 3.** Software tools: versions and sources.

Software tool	Version	Source
BlobToolKit	4.2.1	https://github.com/blobtoolkit/blobtoolkit
BUSCO	5.3.2	https://gitlab.com/ezlab/busco
Hifiasm	0.16.1-r375	https://github.com/chhylp123/hifiasm
HiGlass	1.11.6	https://github.com/higlass/higlass
Merqury	MerquryFK	https://github.com/thegenemyers/MERQURY.FK
MitoHiFi	3	https://github.com/marcelauliano/MitoHiFi
PretextView	0.2	https://github.com/wtsi-hpag/PretextView
purge_dups	1.2.5	https://github.com/dfguan/purge_dups
sanger-tol/genomenote	v1.0	https://github.com/sanger-tol/genomenote
sanger-tol/readmapping	1.1.0	https://github.com/sanger-tol/readmapping/tree/1.1.0
TreeVAL	-	https://github.com/sanger-tol/treeval
YaHS	1.2a.2	https://github.com/c-zhou/yahs

### Wellcome Sanger Institute – Legal and Governance

The materials that have contributed to this genome note have been supplied by a Darwin Tree of Life Partner. The submission of materials by a Darwin Tree of Life Partner is subject to the **‘Darwin Tree of Life Project Sampling Code of Practice’**, which can be found in full on the Darwin Tree of Life website here. By agreeing with and signing up to the Sampling Code of Practice, the Darwin Tree of Life Partner agrees they will meet the legal and ethical requirements and standards set out within this document in respect of all samples acquired for, and supplied to, the Darwin Tree of Life Project. 

Further, the Wellcome Sanger Institute employs a process whereby due diligence is carried out proportionate to the nature of the materials themselves, and the circumstances under which they have been/are to be collected and provided for use. The purpose of this is to address and mitigate any potential legal and/or ethical implications of receipt and use of the materials as part of the research project, and to ensure that in doing so we align with best practice wherever possible. The overarching areas of consideration are:

• Ethical review of provenance and sourcing of the material

• Legality of collection, transfer and use (national and international) 

Each transfer of samples is further undertaken according to a Research Collaboration Agreement or Material Transfer Agreement entered into by the Darwin Tree of Life Partner, Genome Research Limited (operating as the Wellcome Sanger Institute), and in some circumstances other Darwin Tree of Life collaborators.

## Data Availability

European Nucleotide Archive: *Muscardinus avellanarius* (hazel dormouse). Accession number PRJEB64959; https://identifiers.org/ena.embl/PRJEB64959 ( Wellcome Sanger Institute, 2023). The genome sequence is released openly for reuse. The *Muscardinus avellanarius* genome sequencing initiative is part of the Darwin Tree of Life (DToL) project. All raw sequence data and the assembly have been deposited in INSDC databases. The genome will be annotated using available RNA-Seq data and presented through the Ensembl pipeline at the European Bioinformatics Institute. Raw data and assembly accession identifiers are reported in Table 1.

## Author information

Members of the Darwin Tree of Life Barcoding collective are listed here: https://doi.org/10.5281/zenodo.4893703.

Members of the Wellcome Sanger Institute Tree of Life programme are listed here: https://doi.org/10.5281/zenodo.4783585.

Members of Wellcome Sanger Institute Scientific Operations: DNA Pipelines collective are listed here: https://doi.org/10.5281/zenodo.4790455.

Members of the Tree of Life Core Informatics collective are listed here: https://doi.org/10.5281/zenodo.5013541.

Members of the Darwin Tree of Life Consortium are listed here: https://doi.org/10.5281/zenodo.4783558.
