# Supplementary material for: The genome sequence of the hazel dormouse, Muscardinus avellanarius (Linnaeus, 1758)
Source: Wellcome Open Res. 2023 Nov 10;8:514. [Version 1] doi: 10.12688/wellcomeopenres.20360.1 (PMC11190645; doi:10.12688/wellcomeopenres.20360.1)
Supplement: Peer review history

# Reviewer report: Reviewer response for version 1

**DOI:** 10.21956/wellcomeopenres.22546.r72318

**Published:** 2024-08-01

**Authors:** Tyler Scott Alioto

**Affiliations:** 1 Fundacion Centro Nacional de Analisi Genomico, Barcelona, Catalonia, Spain

The genome note submitted by Böhne *et al.* reports the genome sequence of the hazel dormouse, Muscardinus avellanarius The assembly is chromosome-scale and of high quality, meeting the minimum standards recommended by the Earth Biogenome Project. All protocols are appropriate and well-documented. All data conforms with FAIR principles, with read data and assemblies being available in the ENA. Blobtoolkit figures are interactive. Additional QC data are provided at https://tolqc.cog.sanger.ac.uk/, supplementing the figures published in the data note.

Are sufficient details of methods and materials provided to allow replication by others?

Yes

Is the rationale for creating the dataset(s) clearly described?

Yes

Are the datasets clearly presented in a useable and accessible format?

Yes

Are the protocols appropriate and is the work technically sound?

Yes

Reviewer Expertise:

genomics, genome assembly, gene prediction, annotation

I confirm that I have read this submission and believe that I have an appropriate level of expertise to confirm that it is of an acceptable scientific standard.

# Reviewer report: Reviewer response for version 1

**DOI:** 10.21956/wellcomeopenres.22546.r85458

**Published:** 2024-07-16

**Authors:** Richard Edwards

**Affiliations:** 1 Minderoo OceanOmics Centre at UWA, The University of Western Australia Oceans Institute (Ringgold ID: 590767), Perth, Western Australia, Australia

This manuscript presents the genome sequence of the hazel dormouse. The assembly is in very good shape, with high contiguity and chromosome-level scaffolding. It comfortably exceeds the VGP-2020 standards and represents a useful genomic resource for this, and related, organisms. Overall, the presentation of the data is thorough and clear. I only have a few minor comments for consideration.

- **Abstract:** "Most of the assembly is scaffolded into 24 chromosomal pseudomolecules, including the X and Y sex chromosomes."
  - I think this undersells the quality of the genome, which has less than 0.25% unplaced (Table 1).

- **Introduction:** “... and lives arboreally in forests of all types and age classes,”
  - Would it be more accurate to say “temperate forests of all types”? From a global perspective, I don’t think this statement is true. It does not live in rainforests, for example.
- **Page 3:** "Muscardinus avellanarius based on one male specimen from Friesheimer Busch, Germany" - it might be worth clarifying that this is an individual from Lineage 2. (Was this checked?)
- **Page 3, typo:** “... corresponding to the second haplotype have also been deposited.The mitochondrial genome…” - space needed between “deposited.” and “The”.
- **Table 1:** I note that the BUSCO Duplication rate is quite high at 2.9%. Is this typical for the lineage, or is a consequence of having both X and Y in the assembly?
- **Figure 5:** It would be useful to have the chromosomes labeled, as the order in Figure 5 and Table 2 are not the same. The X chromosome has a clear lack of contacts. Is this because it is represented at half-depth in the HiC data? The 3’ end of some of the chromosomes appears to be lacking HiC signal. Is this expected? Some of the chromosome ends also appear to be incorrectly aligned with the HiC signal, e.g. Chromosomes 5, 6, X and 7. This appears to be just a plotting issue, as the interactive map looked fine. Is it possible to fix the plot in the figure?

Are sufficient details of methods and materials provided to allow replication by others?

Yes

Is the rationale for creating the dataset(s) clearly described?

Yes

Are the datasets clearly presented in a useable and accessible format?

Yes

Are the protocols appropriate and is the work technically sound?

Yes

Reviewer Expertise:

Vertebrate and plant genome assembly and curation; Conservation genomics; Evolutionary genomics.

I confirm that I have read this submission and believe that I have an appropriate level of expertise to confirm that it is of an acceptable scientific standard.

# Reviewer report: Reviewer response for version 1

**DOI:** 10.21956/wellcomeopenres.22546.r85452

**Published:** 2024-06-20

**Authors:** Jakub Skorupski

**Affiliations:** 1 Uniwersytet Szczecinski, Szczecin, West Pomeranian Voivodeship, Poland

The article presents a genome assembly of the hazel dormouse *Muscardinus avellanarius*, a species of small rodent found in Europe and Asia Minor. The genome sequence was obtained from a single male specimen collected in Germany. The assembly spans 2,497.5 megabases and includes 24 chromosomal pseudomolecules, covering the X and Y sex chromosomes. The mitochondrial genome has also been assembled. The genome sequence provides significant insights into the genetic makeup of the hazel dormouse and could be instrumental in conservation efforts, given its protected status and the threats it faces due to habitat loss and fragmentation.

 1. Rationale for creating the dataset(s):

 The article convincingly argues for the importance of genomic data in conservation biology, particularly for the hazel dormouse, which faces threats from habitat loss and fragmentation. The genomic data can help in understanding genetic diversity and structuring conservation units, which is crucial for effective conservation strategies. This rationale is well-supported by references to previous studies and the species' conservation status.

 2. Protocols and technical soundness:

 The technical approach is robust, involving high-coverage sequencing and advanced assembly techniques. The use of Pacific Biosciences single-molecule HiFi long reads and chromosome conformation Hi-C data ensures high-quality assembly. The manual curation of the assembly to correct mis-joins further enhances the reliability of the data. The technical soundness is evidenced by the high Quality Value (QV) and the BUSCO completeness score, indicating a comprehensive and accurate assembly.

 3. Details of methods and materials:

 The methods section is detailed, covering all aspects from sample acquisition to DNA extraction and sequencing. The protocols are cited with DOIs, providing easy access to the procedures followed. This level of detail is sufficient for replication, as it includes information on sample preparation, DNA fragmentation, and sequencing technologies used.

 4. Presentation of datasets:

 The datasets are presented in a clear and accessible manner. The article includes figures such as the BlobToolKit Snailplot and GC-coverage plot, which visually summarize the assembly metrics. The data is deposited in public repositories, with accession numbers provided for easy access. The comprehensive presentation ensures that the datasets can be readily used by other researchers.

 The article successfully presents a high-quality genome assembly of the hazel dormouse, providing a valuable resource for conservation biology. The rationale, methods, and data presentation are all robust, making the study a significant contribution to the field. No major revisions are necessary. The article is well-structured and thorough, meeting all criteria for a robust and reproducible genomic study.

Are sufficient details of methods and materials provided to allow replication by others?

Yes

Is the rationale for creating the dataset(s) clearly described?

Yes

Are the datasets clearly presented in a useable and accessible format?

Yes

Are the protocols appropriate and is the work technically sound?

Yes

Reviewer Expertise:

conservation genetics

I confirm that I have read this submission and believe that I have an appropriate level of expertise to confirm that it is of an acceptable scientific standard.

# Reviewer report: Reviewer response for version 1

**DOI:** 10.21956/wellcomeopenres.22546.r70004

**Published:** 2024-01-05

**Authors:** Mostafa Ghaderi-Zefrehei

**Affiliations:** 1 Yasouj University, Yasuj, Kohgiluyeh and Boyer-Ahmad Province, Iran

I am reaching out to you today with a statement of my enthusiastic backing for the manuscript titled "The genome sequence of the hazel dormouse, Muscardinus avellanarius (Linnaeus, 1758)" which was under my review. My perception of this work is that it has been thoughtfully formulated, meticulously executed and highly perceptive. I have recognized how the authors provided noteworthy insights pertaining to our comprehension on this species making it an important contribution. The authors also did an excellent job of discussing their findings in the context of the existing literature. I am confident that this manuscript will be of great interest to readers of Wellcome Open Research. I highly recommend that it be considered for indexing.

 In addition to the strengths mentioned above, and given the fact that I don't consider myself an authority on this particular type of organism, here are some observations regarding your project.

 … “The genome was sequenced from one male Muscardinus avellanarius (Figure 1) collected from Friesheimer Busch, Germany. A total of 58-fold coverage in Pacific Biosciences single-molecule HiFi long reads was generated. Primary assembly contigs were scaffolded with chromosome conformation Hi-C data. Manual assembly curation corrected 34 missing joins or mis-joins, reducing the scaffold number by 6.58%.”…

Strength:

 The utilization of Pacific Biosciences' single-molecule HiFi long reads, which have the ability to cover vast stretches measuring in hundreds of thousands of bases, is a major asset. Its implementation allows for the compilation of intricate sections such as repetitive sequences and

 centromeres that prove daunting for standard short-read sequencing techniques. Another advantage is the integration of chromosome conformation Hi-C data to arrange primary assembly contigs. The Hi-C data records the physical connections among DNA segments, offering useful insights into arranging and aligning contigs on chromosomes.

Cautions/considerations:

 Although it is a typical beginning for genome sequencing initiatives, it's crucial to acknowledge the likelihood of constraints in capturing the genetic variability found among individuals within this species. To attain an all-embracing comprehension of Muscardinus avellanarius' genome, including more study subjects from distinct populations would be advantageous. The completeness of the assembled genome is not directly indicated in the passage. However, it is imperative to evaluate this aspect so as to establish how much of Muscardinus avellanarius' entire genome has been acquired. Such knowledge holds great significance for downstream analyses and interpretations. The crucial step of identifying and characterizing genes as well as other functional elements, referred to as genome annotation, is not discussed for Muscardinus avellanarius. Such an undertaking greatly aids in comprehending the organism's genetic composition and biological processes.

 " … The final assembly has a total length of 2497.5 Mb in 212 sequence scaffolds with a scaffold N50 of 119.5 Mb (Table 1). The snailplot in Figure 2 provides a summary of the assembly statistics, while the distribution of assembly scaffolds on GC proportion and coverage is shown in Figure 3. The cumulative assembly plot in Figure 4 shows curves for subsets of scaffolds assigned to different phyla. Most (99.78%) of the assembly sequence was assigned to 24 chromosomal-level scaffolds, representing 22 autosomes and the X and Y sex chromosomes.

 Chromosome-scale scaffolds confirmed by the Hi-C data are named in order of size (Figure 5; Table 2). While not fully phased, the assembly deposited is of one haplotype. Contigs corresponding to the second haplotype have also been deposited. The mitochondrial genome was

 also assembled and can be found as a contig within the multifasta file of the genome submission ..."

 Although the assembly is presented as a single haplotype, its phasing remains incomplete. To facilitate research on heterozygosity and genome-wide association studies, it's crucial to establish allelic sequence arrangement for each chromosome through phasing.

Strength:

 The confidence in accuracy and contiguity of the assembly is reinforced through chromosome-scale scaffolds validation employing Hi-C data.

Cautions/considerations:

 Although the assembly is deposited as a single haplotype, it lacks complete phasing. Phasing plays a crucial role in researching heterozygosity by determining the sequence of alleles on each chromosome. The passage does not directly mention the thoroughness of the gathered genome.

 Evaluating the comprehensiveness of this assembly is vital in establishing how much of Muscardinus avellanarius's entirety has been encompassed. The passage does not explicitly discuss the extent to which the genome has been compiled comprehensively. To determine how much of Muscardinus avellanarius's entirety has been included, it is crucial to assess the thoroughness of this collection.

Are sufficient details of methods and materials provided to allow replication by others?

Yes

Is the rationale for creating the dataset(s) clearly described?

Partly

Are the datasets clearly presented in a useable and accessible format?

Yes

Are the protocols appropriate and is the work technically sound?

Yes

Reviewer Expertise:

Bioinformatics, Systems Genetics

I confirm that I have read this submission and believe that I have an appropriate level of expertise to confirm that it is of an acceptable scientific standard.
